# Investigation of shared genes and regulatory mechanisms associated with coronavirus disease 2019 and ischemic stroke

**DOI:** 10.3389/fneur.2023.1151946

**Published:** 2023-04-05

**Authors:** Hao Wu, Fei Han

**Affiliations:** ^1^Department of Anesthesiology, Peking Union Medical College Hospital, Peking Union Medical College and Chinese Academy of Medical Science, Beijing, China; ^2^Department of Neurology, Peking Union Medical College Hospital, Peking Union Medical College and Chinese Academy of Medical Science, Beijing, China

**Keywords:** COVID-19, ischemic stroke, protein–protein interaction, weighted correlation network analysis, functional enrichment analysis

## Abstract

**Objective:**

Clinical associations between coronavirus disease (COVID-19) and ischemic stroke (IS) have been reported. This study aimed to investigate the shared genes between COVID-19 and IS and explore their regulatory mechanisms.

**Methods:**

Published datasets for COVID-19 and IS were downloaded. Common differentially expressed genes (DEGs) in the two diseases were identified, followed by protein–protein interaction (PPI) network analysis. Moreover, overlapping module genes associated with the two diseases were investigated using weighted correlation network analysis (WGCNA). Through intersection analysis of PPI cluster genes and overlapping module genes, hub-shared genes associated with the two diseases were obtained, followed by functional enrichment analysis and external dataset validation. Moreover, the upstream miRNAs and transcription factors (TFs) of the hub-shared genes were predicted.

**Results:**

A total of 91 common DEGs were identified from the clusters of the PPI network, and 129 overlapping module genes were screened using WGCNA. Based on further intersection analysis, four hub-shared genes in IS and COVID-19 were identified, including *PDE5A, ITGB3, CEACAM8*, and *BPI*. These hub-shared genes were remarkably enriched in pathways such as ECM-receptor interaction and focal adhesion pathways. Moreover, *ITGB3, PDE5A*, and *CEACAM8* were targeted by 53, 32, and 3 miRNAs, respectively, and these miRNAs were also enriched in the aforementioned pathways. Furthermore, TFs, such as lactoferrin, demonstrated a stronger predicted correlation with the hub-shared genes.

**Conclusion:**

The four identified hub-shared genes may participate in crucial mechanisms underlying both COVID-19 and IS and may exhibit the potential to be biomarkers or therapeutic targets for the two diseases.

## 1. Introduction

Coronavirus disease 2019 (COVID-19) is a highly contagious respiratory disease caused by severe acute respiratory syndrome coronavirus 2 (SARS-CoV-2) (1). Since December 2019, COVID-19 has spread rapidly worldwide, causing considerable morbidity and mortality (2). The clinical manifestations of COVID-19 vary greatly from asymptomatic to severe pneumonia, which can cause respiratory failure and death (3). Due to the rapid evolution of mutant strains of the virus and the difficulties in manufacturing vaccines, it is still highly challenging to fight against COVID-19 (4).

During the pandemic, patients with COVID-19 have demonstrated some neurological complications such as ischemic stroke (IS) (5, 6). IS, also known as cerebral infarction, is a cerebrovascular disease resulting from cerebral blood supply disorder or hemorrhage and is a leading cause of disability and mortality worldwide (7). IS critically affects the quality of life of patients and imposes a huge burden on the public healthcare (8). Patients at a high risk of severe COVID-19 share similar demographics and risk factors with patients at a high risk of IS (9). Moreover, COVID-19 is a risk factor for IS (10, 11). Possible mechanisms include a cytokine storm in response to COVID-19, such as increased levels of C-reactive protein and interleukin (IL)-6, which are associated with an increased risk of IS (12). Additionally, SARS-CoV-2 can increase D-dimer and fibrinogen levels, which are factors that induce IS (13). Furthermore, it may induce hypercoagulability or thrombophilia and increase the formation of blood clots by binding and interacting with the angiotensin-converting enzyme 2 (ACE2) receptors of epithelial and endothelial cells. This is a possible mechanism associated with both COVID-19 and IS (14). In contrast, prior IS is a risk factor for increased severity and mortality associated with COVID-19 (15, 16). Despite this clinical and epidemiological evidence on the relationship between COVID-19 and IS, the shared gene regulation mechanisms remain primarily unknown.

With advances in gene microarray technology and bioinformatics, researchers can quickly detect and analyze the expression data of thousands of genes in a variety of diseases, which can aid in exploring the common pathogenesis of multiple diseases at the genetic level (17, 18). In previous studies, the microarray data GSE16561 and GSE22255 have been used to identify crucial genes associated with IS progression (19, 20). The microarray data GSE157103 and GSE171110 have been used to screen key genes and potential biomarkers for COVID-19 (21, 22). Herein, we downloaded these microarray data for analysis of common differentially expressed genes (DEGs) in COVID-19 and IS and identification of cluster genes using protein–protein interaction (PPI) network analysis. Subsequently, we performed weighted correlation network analysis (WGCNA) to investigate overlapping module genes associated with the two diseases. By intersection analysis of PPI cluster genes and overlapping module genes, hub-shared genes in the two diseases were obtained, followed by functional enrichment analysis and external dataset validation. Moreover, we predicted the upstream micro (mi) RNAs and transcription factors (TFs) of hub-shared genes to elucidate their possible mechanisms. The findings of our study revealed the shared mechanisms of COVID-19 and IS.

## 2. Data and methods

### 2.1. Data acquisition and preprocessing

In this study, eligible datasets were searched and downloaded from the NCBI Gene Expression Omnibus (GEO) database. The following criteria were used to search the GEO datasets: (1) the data format could be analyzed and processed; (2) the data should be clearly classified into disease and control groups; (3) sufficient sample size should be guaranteed, for example, the discovery dataset for two-disease analysis should have at least six samples in each group; and (4) if the above criteria were met, the dataset that could get a more reasonable result was preferable. Finally, the IS-related gene expression profiles GSE16561 and GSE22255, as well as the COVID-19-related gene expression profiles GSE157103 and GSE171110, were selected. The processed probe expression matrix was downloaded directly. Gene symbol transformation was performed based on the corresponding platform annotation file, and the average value of multiple probes corresponding to the same gene symbol was considered as the expression value of the gene. Standardized data were used for subsequent analysis. Among them, GSE16561 and GSE157103 were used as discovery datasets, and GSE22255 and GSE171110 were used as validation datasets.

### 2.2. Differential expression analysis

Based on the expression data of the discovery datasets, the DEGs between patients and controls were identified using the R limma package (version 3.52.2) (23). The value of p was adjusted as the false discovery rate (FDR) using the Benjamini-Hochberg (BH) procedure. The cutoff value was FDR < 0.05 and |log2 fold change (FC)| > 0.263. The intersection of the up-or downregulated genes in the two diseases was considered separately to obtain the common DEGs with consistent up-or downregulation trends associated with the two diseases.

#### 2.2.1. PPI network analysis

Based on the STRING (24) database, a PPI network was constructed using DEGs with consistent up-or down-regulation in the two diseases and visualized using Cytoscape (version 3.9.1) (25). Clusters of the PPI network were analyzed using the MCODE plug-in, and cluster genes were identified.

To reveal the biological function and pathways of cluster genes, ClusterProfiler package (version 4.4.4) (26) was used to analyze the significant Gene Ontology (GO) functions and Kyoto Encyclopedia of Genes and Genomes (KEGG) pathways enriched by these cluster genes. Statistical significance was set at value of *p* <0.05.

### 2.3. WGCNA

Based on the discovery datasets, the gene co-expression networks of IS and COVID-19 were created using the WGCNA package (version 1.71) (27) to obtain the IS and COVID-19-associated modules. The cutoff value was a correlation coefficient (cor) > 0.2 and value of *p* <0.05. The overlapping genes in the IS and COVID-19 modules were considered to be shared genes of the two diseases.

### 2.4. Detection of hub-shared genes in IS and COVID-19

Hub-shared genes of IS and COVID-19 were obtained by intersection analysis of PPI cluster genes and overlapping WGCNA module genes. To understand the biological functions and pathways of these hub-shared genes, we performed GO and KEGG pathway enrichment analyses using the ClusterProfiler package (version 4.4.4). The selection threshold was defined as FDR < 0.05. If there was no significant result when FDR < 0.05 was used as the threshold value, *p* value <0.05 was utilized as the cutoff value for selection.

### 2.5. Validation of hub-shared genes in IS and COVID-19

Using the R limma package (version 3.52.2), differential expression analysis was performed for two validation datasets, GSE22255 and GSE171110. The cutoff value for the GSE22255 dataset was *p* < 0.05, that for GSE171110 was FDR < 0.05, and |log2 FC| > 0.263. DEGs with consistent up-or down-regulation in the two diseases were obtained by intersection analysis of DEGs determined from two validation datasets. Moreover, the differential expression of hub-shared genes was validated using the two validation datasets.

### 2.6. Prediction of upstream miRNAs of hub-shared genes

The upstream miRNAs that could target shared hub genes were predicted using the multiMiR package (28). The miRNA-target gene network was established using Cytoscape (version 3.9.1). To explore miRNA functions, we used the online database MiEAA (29) to perform GO and KEGG pathway enrichment analyses.

### 2.7. TF prediction

To explore the regulatory mechanism of hub-shared genes, their related TFs were predicted using the ChEA3 database (30). Subsequently, a TF gene regulatory network was established using Cytoscape (version 3.9.1).

### 2.8. Statistical analysis

All statistical analyses were performed using the R 4.2.1 package (University of Auckland, New Zealand). Statistical significance was set at value of *p* <0.05.

## 3. Results

### 3.1. Dataset information

Information on the four GEO datasets is summarized in Table 1, including the GSE number, sample size, sample source, and detection platforms. The workflow of this study is shown in Supplementary Figure S1.

**Table 1:** Information of four GEO datasets associated with ischemic stroke and COVID-19.

Group	GSE number	Disease	Sample size	Sample source	Platform
Discovery dataset	GSE16561	Ischemic stroke	39 patients and 24 controls	Whole blood samples	GPL6883 Illumina HumanRef-8 v3.0 expression beadchip
Discovery dataset	GSE157103	COVID-19	100 patients and 26 controls	Plasma and leukocyte samples	GPL24676 Illumina NovaSeq 6,000 (Homo sapiens)
Validation dataset	GSE22255	Ischemic stroke	20 patients and 20 controls	Peripheral blood mononuclear cells (PBMCs)	GPL570 [HG-U133_Plus_2] Affymetrix Human Genome U133 Plus 2.0 Array
Validation dataset	GSE171110	COVID-19	44 patients and 10 controls	Whole blood samples	GPL16791 Illumina HiSeq 2,500 (Homo sapiens)

### 3.2. Identification of DEGs

**Figure 1:**
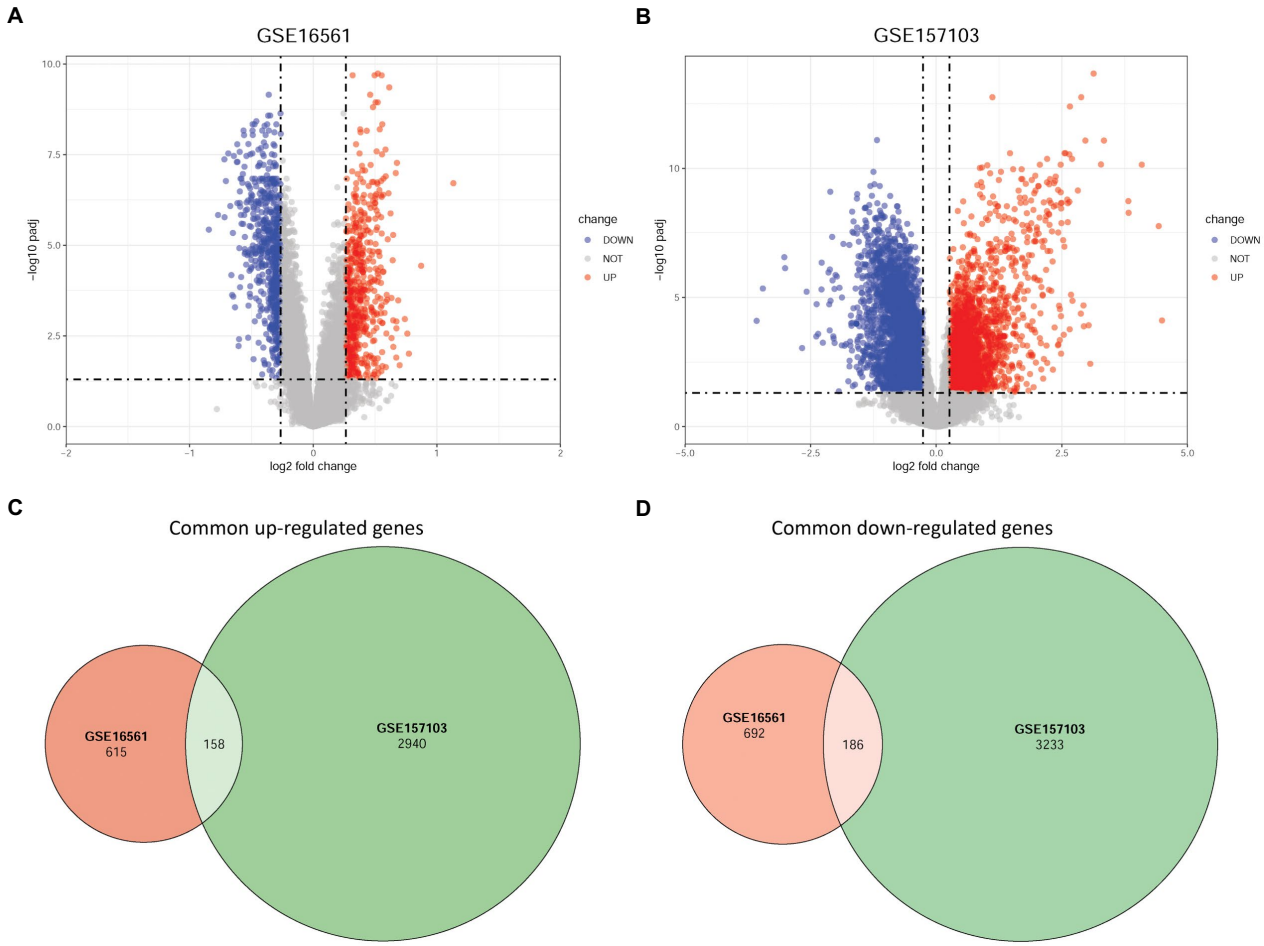
Identification of DEGs based on GSE16561 and GSE157103 datasets. **(A)** Volcano plot of DEGs between IS and normal samples based on the GSE16561 dataset. **(B)** Volcano plot of DEGs between COVID-19 and normal samples based on the GSE157103 dataset. **(C)** Venn diagram of up-regulated DEGs identified based on the two datasets. **(D)** Venn diagram of down-regulated DEGs identified based on the two datasets. DEGs, differentially expressed genes; IS, ischemic stroke; COVID-19, coronavirus disease 2019.

Based on the GSE16561 dataset, 615 upregulated and 692 downregulated DEGs were identified between the IS and normal samples (Figure 1A). Based on the GSE157103 dataset, 2,940 upregulated and 3,233 downregulated DEGs were screened between COVID-19 and normal samples (Figure 1B). Further intersection analysis identified 344 common DEGs with consistent changes in the expression trends associated with the two diseases, including 158 upregulated genes (Figure 1C) and 186 downregulated genes (Figure 1D).

### 3.3. PPI network analysis

**Figure 2:**
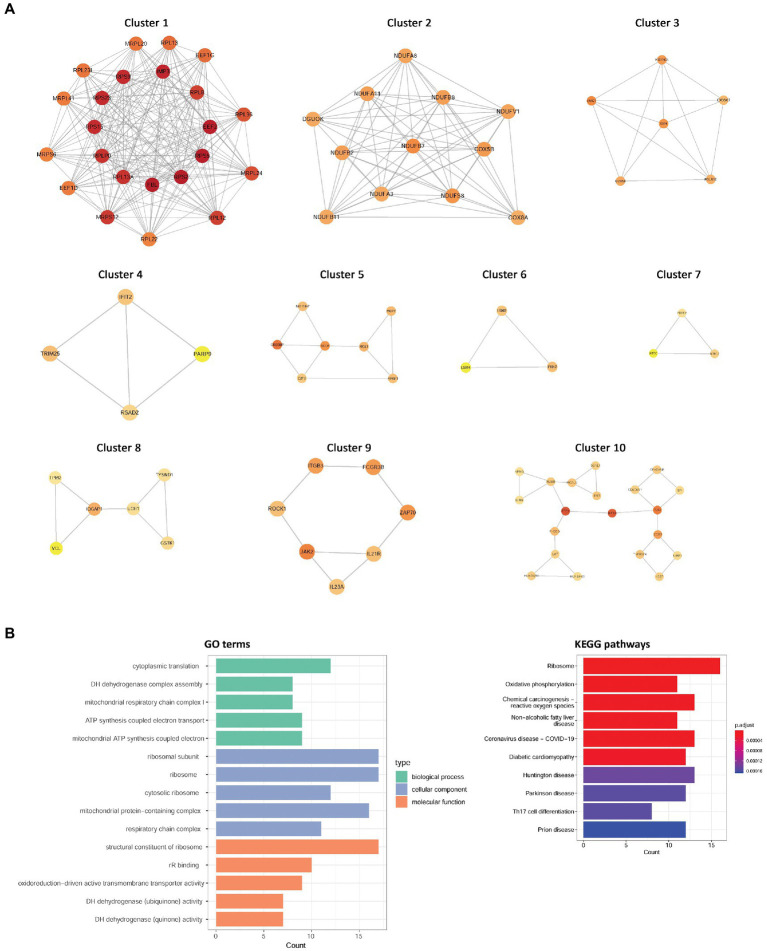
The clusters identified from the PPI network and functional enrichment analysis for cluster genes. **(A)** Ten clusters were identified from this PPI network. **(B)** GO term and KEGG pathway enrichment results. PPI, protein–protein interaction; GO, Gene Ontology; KEGG, Kyoto Encyclopedia of Genes and Genomes.

Using the STRING database, a PPI network including 274 nodes and 1,161 interactions was established by DEGs with consistent up-or downregulation in the two diseases (Supplementary Figure S2). Using the MCODE plug-in in Cytoscape, ten clusters were identified from this PPI network (Figure 2A), comprising 91 genes. Subsequently, these cluster genes were significantly enriched in 218 GO biological process terms (such as cytoplasmic translation and ATP synthesis-coupled electron transport), 62 GO cellular component terms (such as ribosomal subunit and ribosome), 28 GO molecular function terms (such as structural constituents of ribosomes and oxidoreduction-driven active transmembrane transporter activity), and 24 KEGG pathways (such as ribosome and Th17 cell differentiation) (Figure 2B).

### 3.4. Co-expression modules of IS and COVID-19

**Figure 3:**
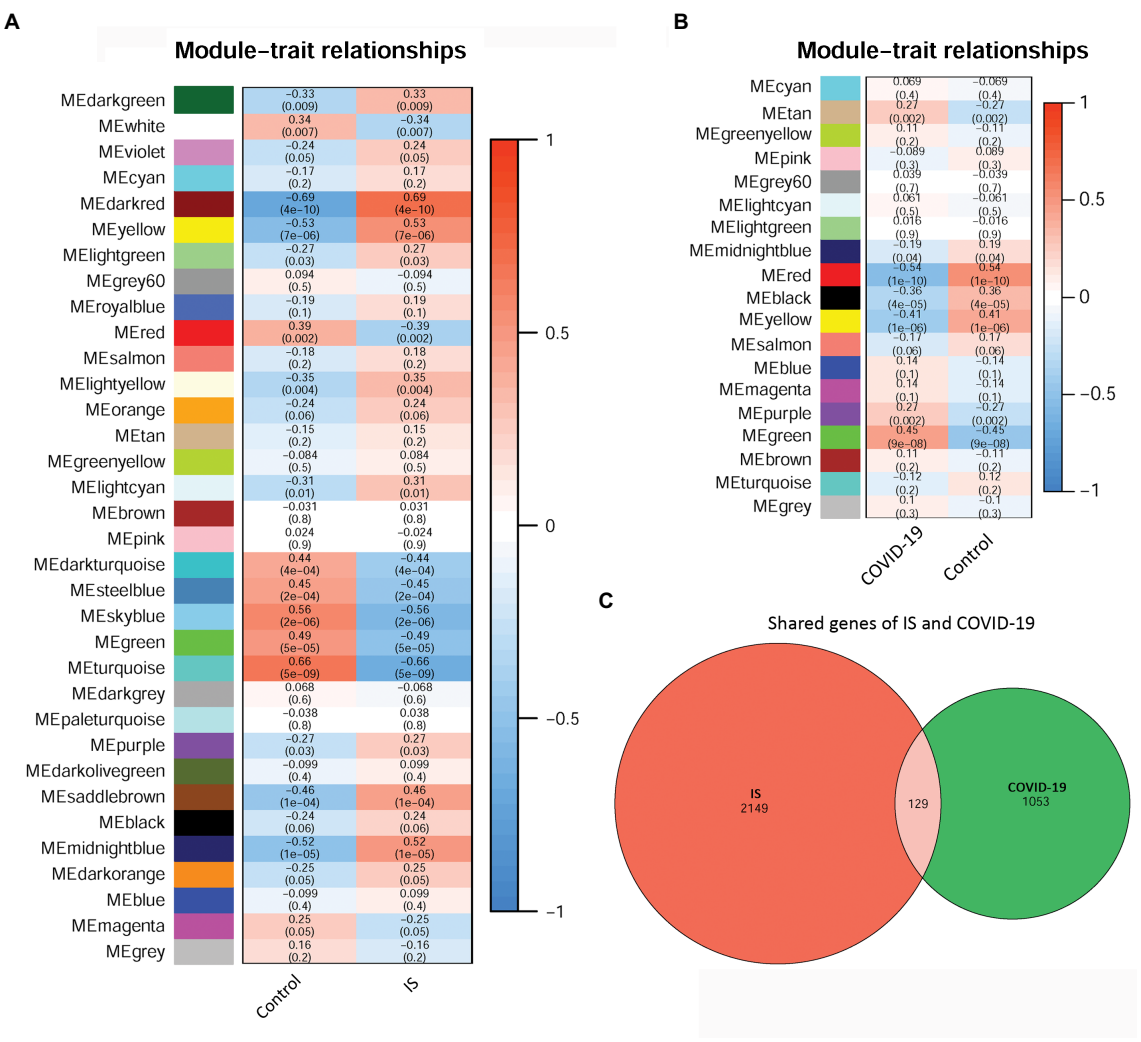
The relationships between WCGNA modules and clinical features of IS and COVID-19. **(A)** Heat map of module–trait relationships in IS. **(B)** Heat map of module–trait relationships in COVID-19. **(C)** Venn diagram showed the shared genes of IS and COVID-19. WCGNA, Weighted correlation network analysis; IS, ischemic stroke; COVID-19, coronavirus disease 2019.

To identify disease-related modules, WGCNA was performed, and the soft power value, β, was set to 6. A total of 10 IS-related modules were identified in GSE16561, including those indicated by dark green, dark red, yellow, light green, light yellow, light cyan, purple, saddle brown, midnight blue, and dark orange (Figure 3A). Additionally, three COVID-19-related modules were screened in GSE157103, including those indicated by tan, purple, and green (Figure 3B). By further intersection analysis, 129 overlapping module genes in the IS and COVID-19 modules were obtained, which were considered to be the shared genes of the two diseases (Figure 3C).

### 3.5. Detection of hub-shared genes in IS and COVID-19

**Figure 4:**
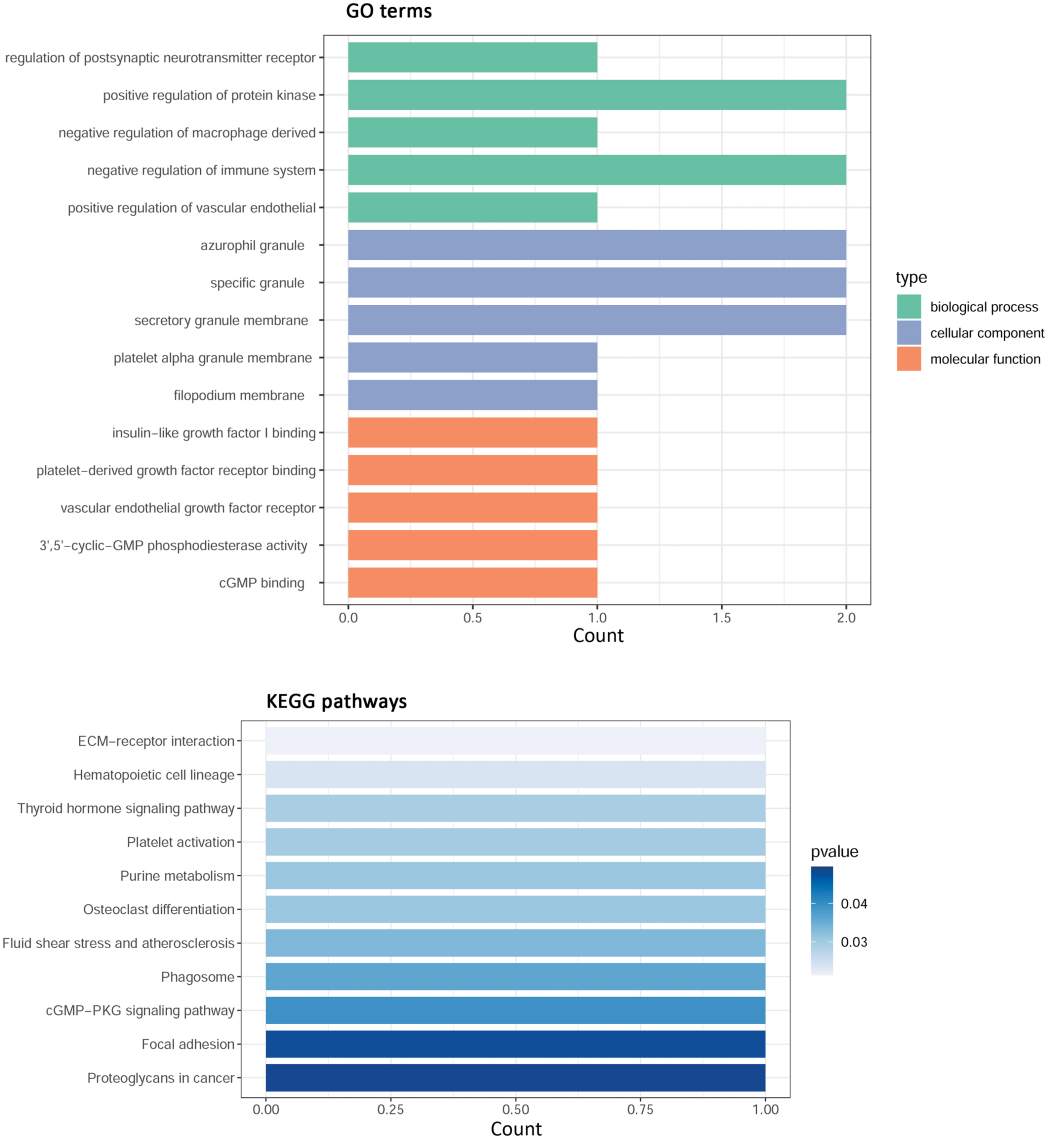
Functional enrichment analysis for hub shared genes in IS and COVID-19. IS, ischemic stroke; COVID-19, coronavirus disease 2019; GO, Gene Ontology; KEGG, Kyoto Encyclopedia of Genes and Genomes.

By intersection analysis of PPI cluster genes and overlapping WGCNA module genes, four hub-shared genes in IS and COVID-19 were obtained, including phosphodiesterase 5A (PDE5A), integrin subunit beta 3 (ITGB3), CEA cell adhesion molecule 8 (CEACAM8), and bactericidal permeability increasing protein (BPI). These hub-shared genes were remarkably enriched in 31 GO biological process terms (such as regulation of postsynaptic neurotransmitter receptor and negative regulation of the immune system), 17 GO cellular component terms (such as azurophil granules and specific granules), 20 GO molecular function terms (such as insulin-like growth factor I binding and platelet-derived growth factor receptor binding), and 11 KEGG pathways (such as ECM-receptor interaction and focal adhesion; Figure 4).

### 3.6. Validation of hub-shared genes in IS and COVID-19

**Figure 5:**
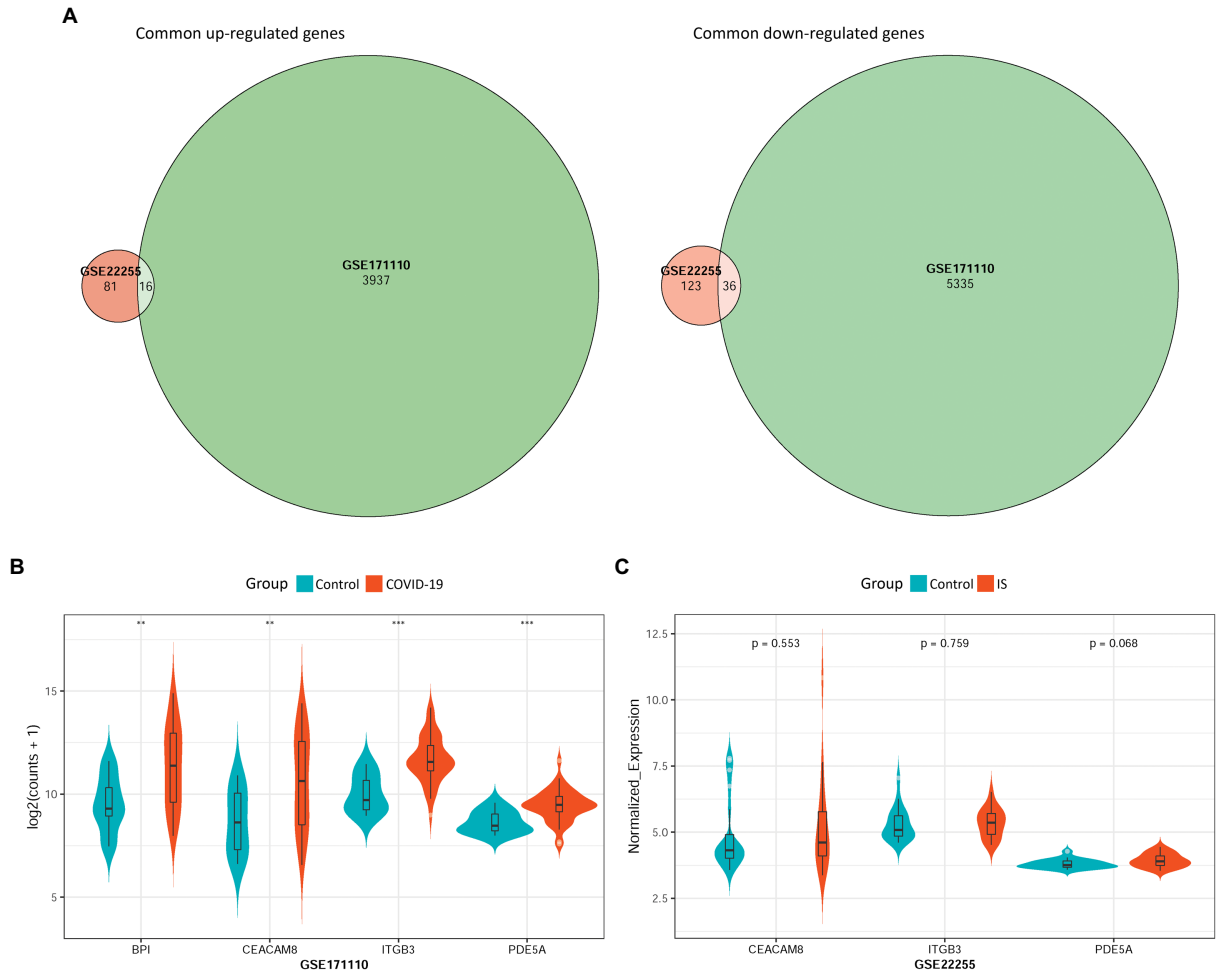
Validation of hub-shared genes in IS and COVID-19 using two external validation datasets GSE22255 and GSE171110. **(A)** Common DEGs with consistent changes in the expression trends in the two diseases, including 16 up-regulated genes and 36 down-regulated genes. **(B)** The differential expression of four hub-shared genes (PDE5A, ITGB3, CEACAM8, and BPI) in the GSE171110 dataset. **(C)** The differential expression of hub-shared genes in the GSE22255 dataset. IS: ischemic stroke; COVID-19: coronavirus disease 2019.

To validate the hub-shared genes, differential expression analysis was performed for two validation datasets: GSE22255 and GSE171110. A total of 256 (97 upregulated and 159 downregulated) DEGs and 9,324 (3,953 upregulated and 5,371 downregulated) DEGs were obtained based on the GSE22255 and GSE171110 datasets, respectively. Intersection analysis revealed 52 DEGs with consistent changes in the expression trends associated with the two diseases, including 16 upregulated and 36 downregulated genes (Figure 5A). Moreover, the differential expression of the four hub-shared genes (*PDE5A, ITGB3, CEACAM8*, and *BPI*) was validated in the two validation datasets. The results showed that the four hub-shared genes were all significantly up-regulated in COVID-19 samples compared to the expression in control samples in the GSE171110 (Figure 5B). However, no expression value of the *BPI* gene was determined, and no significant differences were detected in the expression of the other three genes between the IS and control samples in the GSE22255 dataset (Figure 5C).

### 3.7. Analysis of upstream miRNAs of hub-shared genes

**Figure 6:**
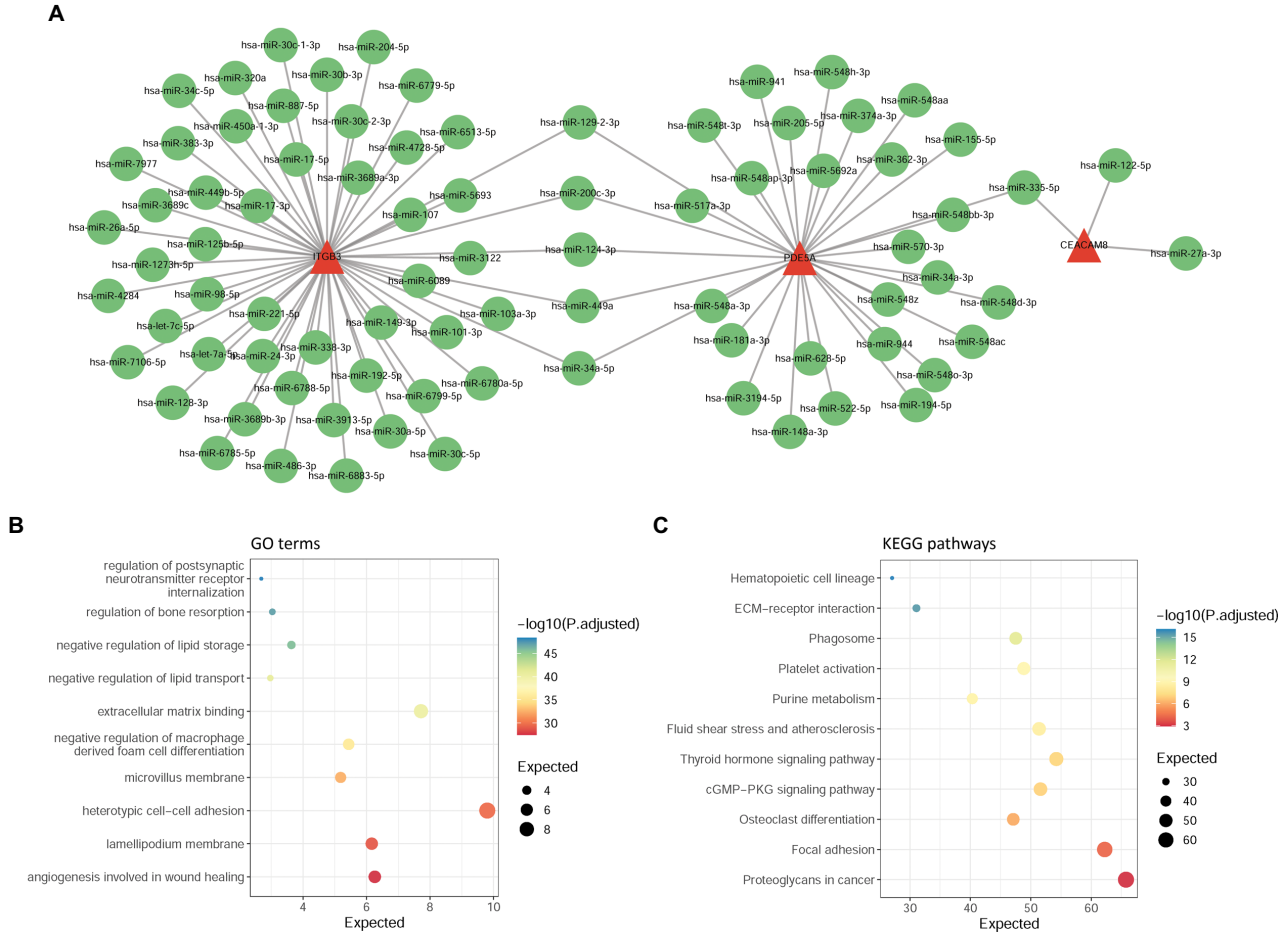
Analysis of upstream miRNAs of hub-shared genes. **(A)** The miRNA-target network constructed by four hub-shared genes and their predicted miRNAs. **(B)** GO enrichment results for upstream miRNAs of hub shared genes. **(C)** KEGG pathway enrichment results for upstream miRNAs of hub-shared genes. GO, Gene Ontology; KEGG, Kyoto Encyclopedia of Genes and Genomes.

To explore the regulatory mechanism of hub-shared genes, we used the multiMiR package to predict the upstream miRNAs of four hub-shared genes, and a total of 82 miRNAs were determined. An miRNA-target network was constructed (Figure 6A). In this network, *ITGB3, PDE5A*, and *CEACAM8* were targeted by 53, 32, and 3 miRNAs, respectively. For example, *ITGB3* and *PDE5A* could be targeted by miRNAs such as miR-200c-3p and miR-34a-5p, and *PDE5A* and *CEACAM8* could be targeted by miR-335-5p. No upstream miRNAs were determined for *BPI*. Subsequent functional enrichment analysis for these predicted upstream miRNAs indicated that 68 GO terms, such as regulation of postsynaptic neurotransmitter receptors and regulation of bone resorption, and 11 KEGG pathways, such as ECM-receptor interaction and focal adhesion, were identified to be the same as those enriched by the hub-shared genes. The top 10 GO terms and KEGG pathways are shown in Figures 6B,C, respectively.

### 3.8. TF prediction analysis

**Figure 7:**
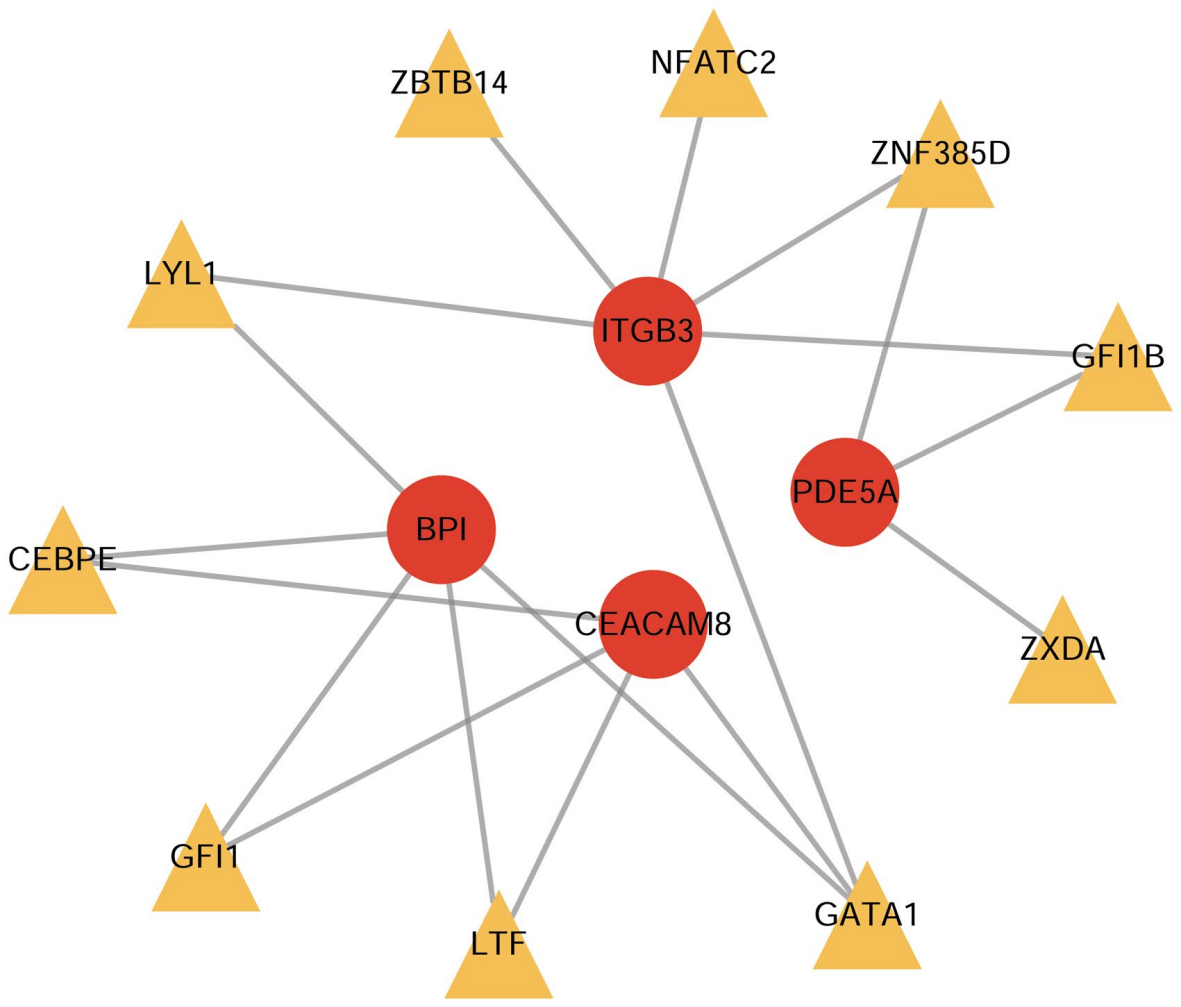
The TF-gene regulatory network constructed by hub shared genes and the top 10 predicted TFs. TF, transcription factor.

Based on the ChEA3 database, we predicted the TFs of hub-shared genes. The top 10 TFs with stronger predicted correlations were lactoferrin (LTF), zinc finger protein 385D (ZNF385D), GATA binding protein 1 (GATA1), CCAAT enhancer binding protein epsilon (CEBPE), nuclear factor of activated T cells 2 (NFATC2), growth factor independent 1 transcriptional repressor (GFI1), zinc finger X-linked duplicated A (ZXDA), zinc finger and BTB domain-containing 14 (ZBTB14), growth factor independent 1 B transcriptional repressor (GFI1B), and LYL1 basic helix–loop–helix family member (LYL1). The TF gene regulatory network constructed using the four hub-shared genes and the top 10 TFs is shown in Figure 7.

## 4. Discussion

In the present study, we explored the hub-shared genes of COVID-19 and IS and their regulatory mechanisms by a series of bioinformatics analyses of disease-related GEO datasets. We found that *PDE5A*, *ITGB3*, *CEACAM8*, and *BPI* were hub-shared genes in IS and COVID-19, which were remarkably enriched in pathways such as ECM-receptor interaction and focal adhesion pathways. Moreover, *ITGB3*, *PDE5A*, and *CEACAM8* were targeted by 53, 32, and 3 miRNAs, respectively, and these miRNAs were also enriched in the aforementioned pathways. Furthermore, TFs, such as lactoferrin, demonstrated a stronger predicted correlation with the hub-shared genes. These results discover the potential common mechanisms underlying two diseases.

COVID-19 presents a major public health challenge owing to its highly contagious nature and large number of deaths worldwide. Acute IS associated with COVID-19 is a deadly condition that cannot be ignored. An increasing number of studies have suggested a link between COVID-19 and IS (31–33). However, the shared molecular mechanisms underlying these two diseases have not yet been fully elucidated. Herein, to screen hub-shared genes in COVID-19 and IS, we first analyzed common DEGs in IS and COVID-19 samples and further screened DEGs in the clusters of the PPI network. We then explored the common genes associated with IS and COVID-19 using WGCNA, which can identify clinical trait-related genes and obtain co-expression modules with high biological significance (27). By intersecting DEGs in the PPI clusters and WGCNA module genes, we obtained four hub-shared genes in IS and COVID-19, including *PDE5A, ITGB3, CEACAM8*, and *BPI*. PDE5A is a cGMP-specific phosphodiesterase that plays a key role in regulating smooth muscle relaxation in the cardiovascular system (34). PDE5A has been identified as a key target of anti-stroke traditional Chinese medicinal compounds (35). ITGB3 is an adhesion molecule that promotes platelet aggregation and blood clot development (36). *ITGB3* is a key gene involved in the development of COVID-19-related stroke (37), in line with our findings. CEACAMs are cell adhesion molecules that participate in diverse tissue-dependent functions such as adhesion regulation, lymphocyte activation, and neutrophil activation (38). CEACAM8 is a marker of neutrophil activation and specific granules in neutrophils (39). *BPI* is also expressed in the granules of human neutrophils. Neutrophils are key players in the pathophysiology of both COVID-19 (40) and IS (41). Given the biological function of these hub-shared genes, we believe that these genes might be crucial regulators in the development of these two diseases and serve as potential therapeutic targets.

Strikingly, cardiovascular risk factors such as hypertension, dyslipidemia, and diabetes mellitus are modifiers of the interaction between pathogenic mechanisms and stroke (42). *PDE5A* is a hypertension-related gene that regulates cardiac tone and vascular function (43). There is a relationship between diabetes mellitus and *PDE5A* polymorphism regarding the response to sildenafil treatment (44). *CEACAM8* is reported to be associated with atherosclerosis and type 2 diabetes (45). In addition, there are some co-morbidities of stroke, such as peripheral artery disease and coronary artery disease (CAD). A previous study revealed an association of PDE5A variant and CAD (46). High frequency of the *ITGB3* C allele was observed in CAD patients (47). These data suggested that these stroke risk factors and co-morbidities may affect the development of COVID-19 and IS *via* regulating these hub-shared genes. Despite these, whether stroke risk factors and co-morbidities contribute to the dysregulation of hub-shared genes are required to be investigated in the future.

To better understand the possible mechanism of hub-shared genes in COVID-19 and IS, we conducted functional enrichment analysis. These hub-shared genes were implicated in key pathways such as ECM-receptor interaction and focal adhesion. ECM-receptor interaction is related to inflammatory responses after stroke (48). Focal adhesion is associated with adhesion and migration of vascular smooth muscle cells, which play a key role in stroke (49). ECM-receptor interactions and focal adhesions are also associated with COVID-19 (50). These pathways may be associated with key mechanisms mediating the function of hub-shared genes in the development of COVID-19 and IS.

miRNAs are post-transcriptional regulators that regulate the expression of their target genes. miRNAs play important roles in COVID-19 (51) and IS (52). We further discovered a link between COVID-19 and IS in terms of miRNA expression. In the miRNA-target network, *ITGB3, PDE5A*, and *CEACAM8* were targeted by 53, 32, and 3 miRNAs, respectively. For instance, ITGB3 and PDE5A are targeted by a large number of miRNAs, such as miR-200c-3p and miR-34a-5p. miR-200c-3p expression is related to COVID-19 and may be a predictor of COVID-19 severity (53). Peripheral blood levels of miR-200c-3p have been suggested as potential biomarkers for IS (54). The reduced miR-34a-5p expression has been observed in lung tissues and airway samples of patients with COVID-19 (55, 56). Increased miR-34a-5p expression induces brain cell apoptosis in patients with acute IS (57). Moreover, *PDE5A* and *CEACAM8* were targeted by miR-335-5p. The levels of miR-335-5p are reduced in plasma samples of patients with IS (58). Circ_0101874 overexpression can promote neuronal injury in IS by regulating the miR-335-5p/PDE4D axis (59). Meanwhile, miR-335-5p is downregulated in patients with COVID-19 relative to its expression in patients with community-acquired pneumonia (60). Furthermore, these predicted miRNAs of hub-shared genes were also enriched in key pathways, such as ECM-receptor interaction and focal adhesion, which were the same as those enriched in association with the hub-shared genes. Therefore, these miRNAs may also be key regulators of COVID-19 and IS.

TFs play an important role in regulating gene expression. We explored the relationships between TFs and hub-shared genes. Based on the TF–gene interaction network, TFs, such as LTF, showed stronger predicted correlations with hub-shared genes. LTF is an iron-binding glycoprotein of the transferrin family with anti-inflammatory, antioxidant, antibacterial, and antiviral properties (61). LTF improves neurological deficits associated with stroke (62). Accumulating evidence has revealed the potential applications of LFT in the COVID-19 management (63–65). Targeting LTF may be a treatment strategy for COVID-19 and IS. In addition to LTF, other TFs, such as ZNF385D, GATA1, CEBPE, NFATC2, GFI1, ZXDA, ZBTB14, GFI1B, and LYL1, showed strong predicted correlations with hub-shared genes. The functions of these TFs in COVID-19 and IS warrant further investigation.

This study has several limitations that should not be neglected. First, these results, including hub-shared genes, key pathways, candidate miRNAs, and TFs, were obtained from bioinformatics analyses of publicly available datasets. Second, the expression of the four hub-shared genes did not show a significant difference between IS and control samples in the validation dataset, which may be attributed to the sample size. No other datasets were available for validation. Last, the genetic architectures of diseases are complex, and it is difficult to judge the validity of one method over another during gene study. Overall, the function and clinical applications of hub-shared genes in COVID-19 and IS should be validated using additional basic experiments or clinical trials.

In conclusion, our findings reveal that the four identified hub-shared genes may be associated with crucial mechanisms underlying both COVID-19 and IS and may exhibit the potential as biomarkers or therapeutic targets for two diseases.

## Data availability statement

The original contributions presented in the study are included in the article/Supplementary material, further inquiries can be directed to the corresponding author.

## Ethics statement

Ethical review and approval was not required for the study on human participants in accordance with the local legislation and institutional requirements. Written informed consent from the patients/participants or patients/participants’ legal guardian/next of kin was not required to participate in this study in accordance with the national legislation and the institutional requirements.

## Author contributions

HW contributed to data analysis and manuscript drafting. FH revised the data for the intellectual contents and responsible for study conception and interpretation. All authors contributed to the article and approved the submitted version.

## Funding

The study was funded by the National High Level Hospital Clinical Research Funding (grant number: 2022-PUMCH-A-068), and the National Natural Science Foundation of China (grant number: 82271368).

## Conflict of interest

The authors declare that the research was conducted in the absence of any commercial or financial relationships that could be construed as a potential conflict of interest.

## Publisher’s note

All claims expressed in this article are solely those of the authors and do not necessarily represent those of their affiliated organizations, or those of the publisher, the editors and the reviewers. Any product that may be evaluated in this article, or claim that may be made by its manufacturer, is not guaranteed or endorsed by the publisher.

## Supplementary material

SUPPLEMENTARY FIGURE S1 The workflow of this study.

SUPPLEMENTARY FIGURE S2 The PPI network constructed by DEGs with consistent up- or down regulation in the two diseases. PPI: protein-protein interaction; DEGs: Deferentially expressed genes.
