# Supplementary figures and images for: Investigation of shared genes and regulatory mechanisms associated with coronavirus disease 2019 and ischemic stroke
Source: Front Neurol. 2023 Apr 5;14:1151946. doi: 10.3389/fneur.2023.1151946 (PMC10115163; doi:10.3389/fneur.2023.1151946)

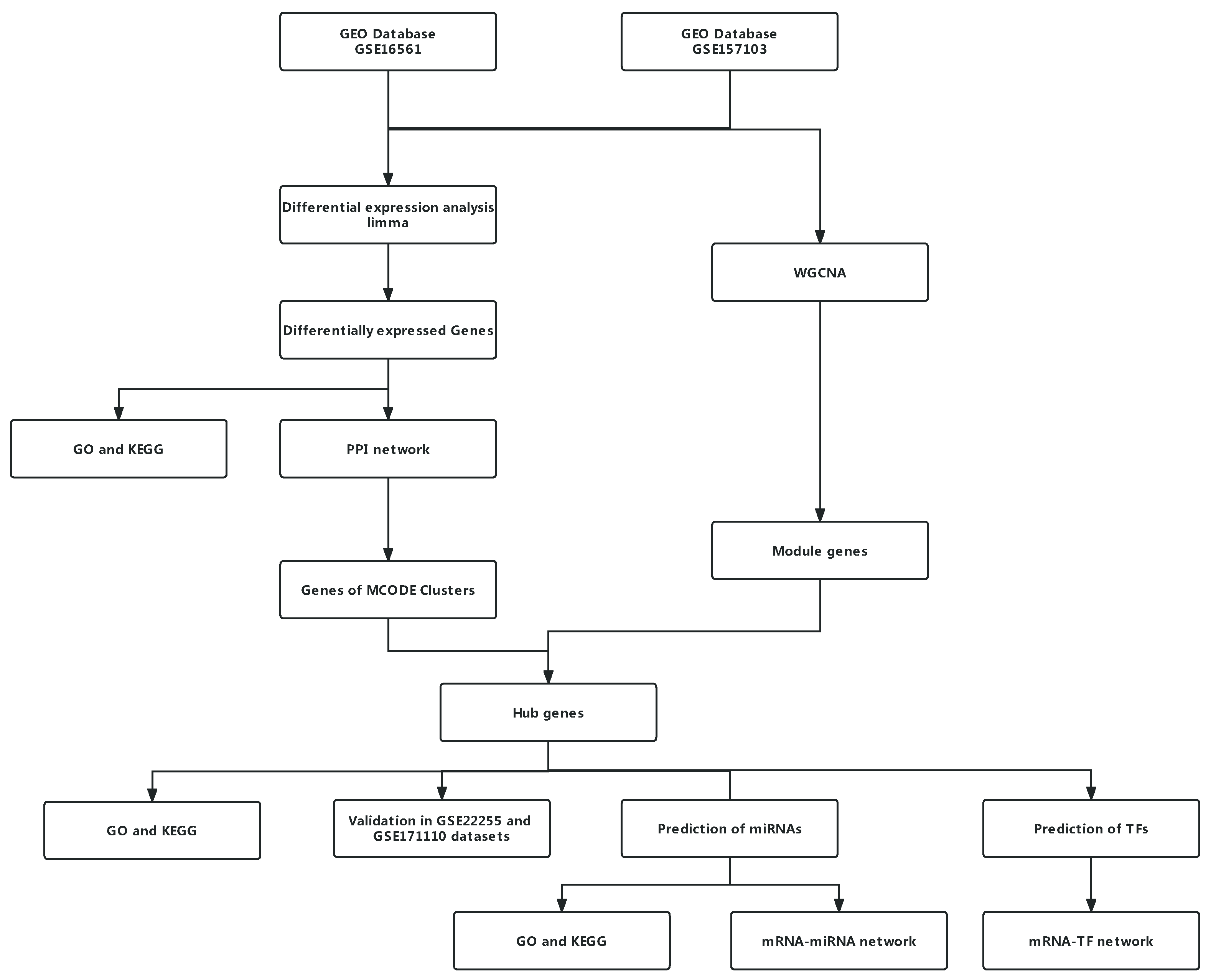

Supplement: SUPPLEMENTARY FIGURE S1 — The workflow of this study. [file Image_1.TIF]

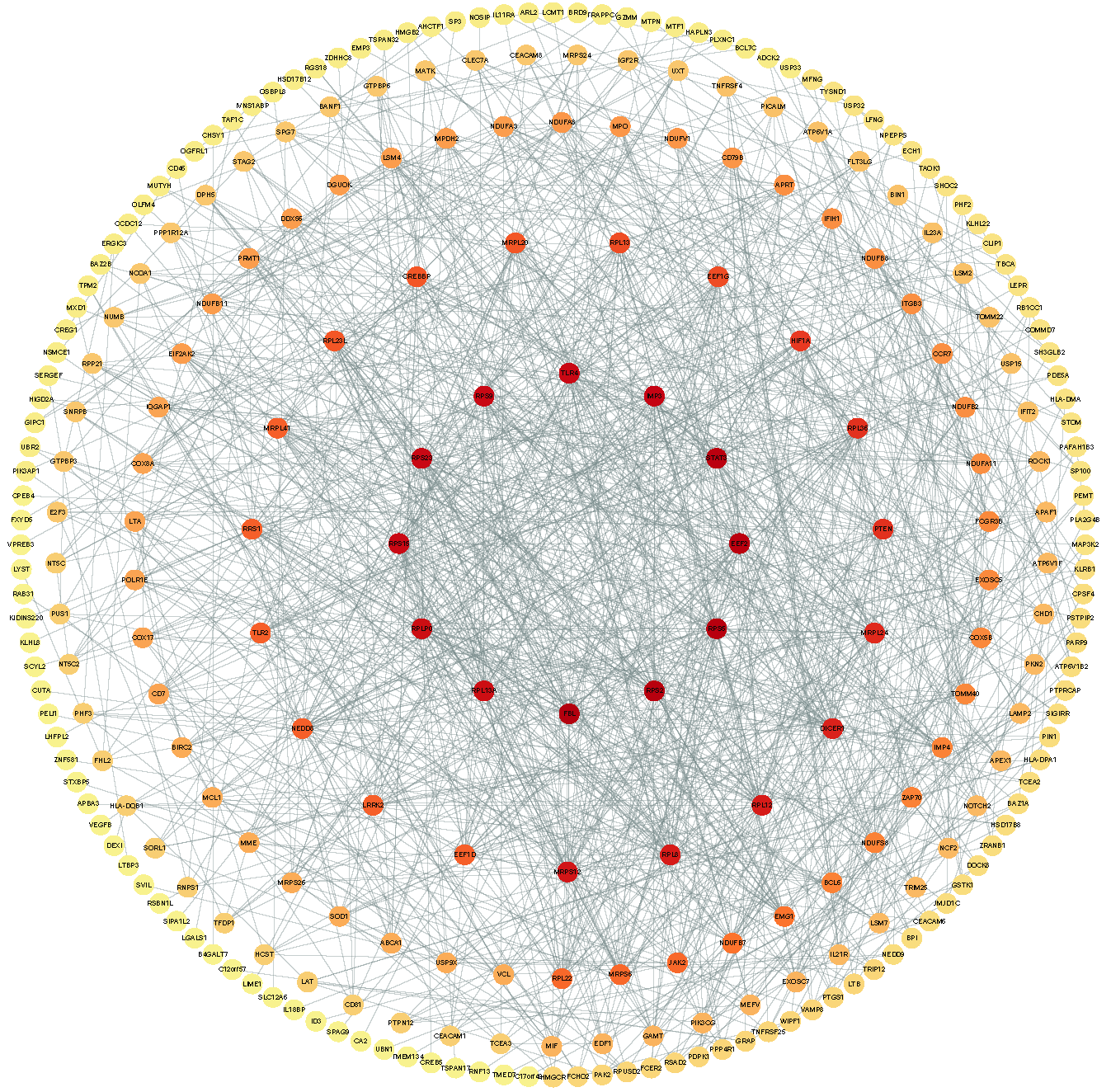

Supplement: SUPPLEMENTARY FIGURE S2 — The PPI network constructed by DEGs with consistent up- or down regulation in the two diseases. PPI: protein-protein interaction; DEGs: Deferentially expressed genes. [file Image_2.TIF]
